# Pulmonary Arteriovenous Malformation (AVM) Causing Tension Hemothorax in a Pregnant Woman Requiring Emergent Cesarean Delivery

**DOI:** 10.1155/2011/865195

**Published:** 2011-06-02

**Authors:** Nidhi Sood, Nikhil Sood, Vibhu Dhawan

**Affiliations:** ^1^OSF Saint Francis Medical Center, Peoria, IL 61637, USA; ^2^GB Pant Hospital, New Delhi 110016, India; ^3^College of Medicine, University of Illinois, Peoria, IL, USA

## Abstract

Pulmonary arteriovenous malformations (PAVMs), although most commonly congenital, are usually detected later in life. We present a case of a 25-year-old woman with no previous history of AVM or telangiectasia, who presented with life-threatening hypoxia, hypotension, and pleuritic chest pain in 36th week of gestation. Chest tube placement revealed 4 liters of blood. Patient was subsequently found to have bleeding pulmonary AVM as the source of hemothorax. Successful embolisation of the bleeding vessel followed by thoracoscopic evacuation of the organized clot relieved the hypoxia. Further screening for AVM revealed large splenic AVM for which patient underwent splenectomy in the coming months.

## 1. Case Presentation

Our patient was a 25-year-old previously healthy female at 36th week gestation presented to the hospital with 4 days of progressive dyspnea and right-sided pleuritic chest pain. Her past medical history was pertinent for gestational thrombocytopenia which resolved spontaneously but no history of epistaxis or family history of Osler-Weber-Rendu syndrome was noted. She was found to be hypotensive and hypoxic on ventilator support. Chest X-ray revealed right-sided pleural effusion which caused complete hemithorax opacification.

Patient underwent emergent cesarean delivery due to unstable vital signs and frequent fetal decelerations. Due to persistent hypoxia and hypotension, patient was admitted to ICU with ventilator support. Physical exam was significant for decreased breath sounds on the right side, dull percussion note, and decreased vocal tactile fremitus but no evidence of cyanosis. 

Vital signs were systolic blood pressure of 80 mm Hg, diastolic blood pressure of 40 mm Hg, pulse rate 142/min, pulse oximetry saturation 86% to 88% on 100% inspired oxygen, afebrile, and respiratory rate of 30/min. Patient was visibly diaphoretic using accessory muscles with sinus tachycardia noted on monitor. 

Initial labs revealed normal platelets, normal coagulation panel, and hemoglobin of 6.5 gm/dL. Critical care panel showed pH of 7.4, pCO2 of 50 mm hg, pAO2 60 mm hg, and saturation of 88%. 

**Figure 1:**
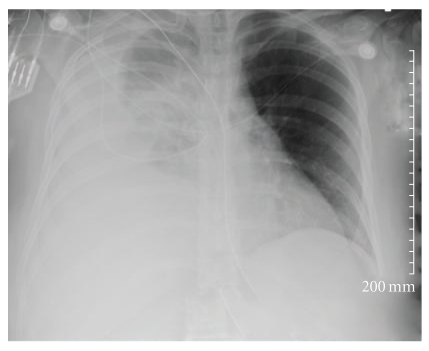
Chest X-ray showing right hemithorax opacification.

A chest tube was placed which revealed a hemothorax. four liters of frank blood was removed with the chest tube placement which also resulted in normalization of the blood pressure and improved oxygenation on the monitor. Repeat chest X-ray showed continued opacification of the right lung, and the patient's hemoglobin continued to fall (Figure 1). CT-chest with IV contrast showed a likely 1 cm area of active contrast extravasation along with compressive atelectasis of the right lower lobe. 

Pulmonary angiography confirmed an AVM with feeding branch of a basilar right pulmonary artery supplying aneurysmal AVM and dilated draining vein. Embolisation of the culprit vessel was performed (Figure 3).

Repeat chest X-ray showed continued opacification of the right lung and suspecting an organized blood clot thoracoscopic evacuation was performed.

## 2. Results

Patient had an uncomplicated course subsequently and was discharged home after 4 days. Evaluation for AVM with a head MRI was negative. CT scan of the abdomen revealed splenomegaly with a large 11 cm × 9 cm × 6 cm splenic mass. Patient then underwent splenectomy, where the final pathology report showed the splenic mass consistent with AVM. Examination or genetic testing for hereditary hemorrhagic telangiectasia (HHT) was not performed during this hospitalization. 

## 3. Discussion

Pulmonary AVMs are abnormal vascular structures that provide a direct capillary-free communication between the pulmonary and systemic circulation which causes right-to-left shunt, hypoxia, and increases the risk of paradoxical emboli including cerebral abscess [1, 2]. 

These AVMs increase in size during pregnancy [3]. 

PAVMs occur most commonly in individuals affected by the inherited vascular disorder HHT, or Osler-Weber-Rendu syndrome [4, 5]. In a series of 219 consecutive PAVM patients, a clinical diagnosis of HHT could be established in 93.6% of cases [2]. Criteria for hereditary hemorrhagic telangiectasia include spontaneous recurrent nosebleeds, mucocutaneous telangiectasia, visceral involvement, and an affected first-degree relative [5]. It is important for families (and medical practitioners) to be aware that no child of a patient with HHT can be informed that he or she does not have HHT unless a genetic test has been performed for a disease causing mutation, as such a mutation can be identified in 85% of HHT patients [6]. 

It is important to evaluate all patients with PAVM for signs of HHT. This involves meticulous exam for telangiectatic lesions by looking in the oral cavity, the lips, and nasal mucosa using rigid rhinoscopy. The skin of the face and fingertips and look in the conjunctive of the eye should be included. 

Criteria for screening for AVM include patients with HHT patients with cerebral abscesses and any young patient with an embolic cerebrovascular accident even if an apparent alternative cause is present [7]. 

**Figure 2:**
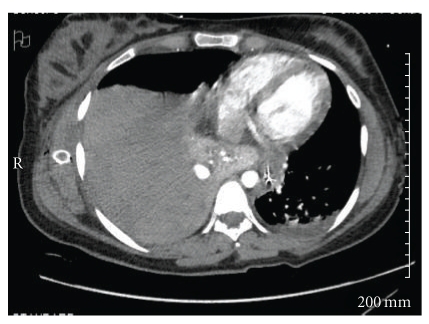
Computed tomography of the chest with I.V. contrast showing right-sided pleural effusion.

**Figure 3:**
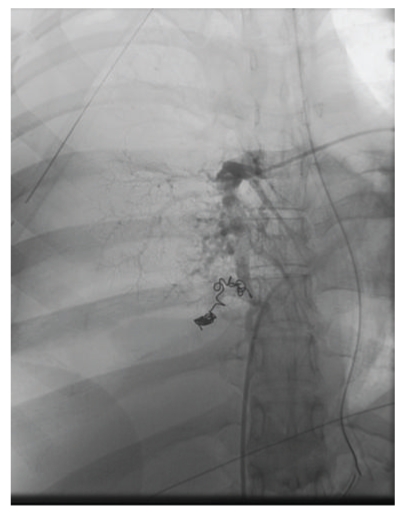
Interventional radiologist-guided embolisation of the right pulmonary artery which was the culprit vessel.

Screening for pulmonary AVM includes chest radiograph, arterial blood gas on oxygen, and bubble contrast echocardiogram to evaluate for pulmonary shunting [8]. Screening for cerebral AVM includes MRI and MR angiography (MRA) of the brain although cerebral angiography is most sensitive. Computed tomography of the abdomen and pelvis is recommended for screening for AVM in the abdomen (Figure 2) [8]. 

Repeat screening procedures every 5 years or at times when the number and size of AVM increase such as during puberty or pregnancy [9]. 

Pregnancies should be managed with close liaison between obstetricians, pulmonologists, and interventional radiologists, using appropriate “high-risk” obstetric management strategies [9]. To reduce risk of brain abscess in cases of undiagnosed pulmonary AVM, antibiotic prophylaxis is recommended prior to dental procedures [10]. Embolisation remains the treatment of choice as demonstrated by multiple studies [11]. 

## 4. Conclusion

Previously, several cases of pulmonary AVM have been described in the literature with a large proportion of cases associated with hereditary telangiectasia. 

Our patient's presentation with life-threatening findings in pregnancy with no previously significant medical history is unique. These AVM expand during pregnancy because of increases in blood volume, cardiac output, and venous distensibility [3]. Presence of AVM should be considered in the setting of pregnancy, hypoxia, and spontaneous hemothorax. Further screening for other sites of AVM should be discussed and pursued. Women with known pulmonary AVM should be maximally treated prior to becoming pregnant, and the physician should be alert to complications of pulmonary AVM during pregnancy. This case emphasizes the importance of thinking beyond the current event of hemothorax which resulted in finding of large splenic AVM in timely manner, and subsequent treatment prevented complications. Examination or genetic testing for hereditary hemorrhagic telangiectasia (HHT) was regrettably not performed.
